# Supplementary material for: Reward and aversion processing by input-defined parallel nucleus accumbens circuits in mice
Source: Nat Commun. 2022 Oct 21;13:6244. doi: 10.1038/s41467-022-33843-3 (PMC9587247; doi:10.1038/s41467-022-33843-3)
Supplement: Supplementary file 1 — Supplementary Information [file 41467_2022_33843_MOESM1_ESM.docx]

**Reward and aversion processing by input-defined parallel**

**nucleus accumbens circuits in mice**

Kuikui Zhou^1,2,#^, Hua Xu^1,#^, Shanshan Lu^1,3,#^, Shaolei Jiang^1,4^, Guoqiang Hou^1^, Xiaofei Deng^1^, Miao He^5^, Yingjie Zhu^1,3,6,7,8,*^

^1^ Shenzhen Key Laboratory of Drug Addiction, Shenzhen Neher Neural Plasticity Laboratory, the Brain Cognition and Brain Disease Institute, Shenzhen Institute of Advanced Technology, Chinese Academy of Sciences; Shenzhen-Hong Kong Institute of Brain Science-Shenzhen Fundamental Research Institutions, Shenzhen, 518055, China.

^2^ School of Health and Life Sciences, University of Health and Rehabilitation Sciences, Qingdao, 266071, China.

^3^ University of Chinese Academy of Sciences, Beijing, 100049, China.

^4^ University of Shanghai for Science and Technology, Shanghai, 200093, China.

^5^ Institutes of Brain Science, Department of Neurology, State Key Laboratory of Medical Neurobiology and MOE Frontiers Center for Brain Science, Zhongshan Hospital, Fudan University, Shanghai, 200032, China

^6^ Faculty of Life and Health Sciences, Shenzhen Institute of Advanced Technology, Chinese Academy of Sciences, Shenzhen, 518055, China.

^7^ CAS Key Laboratory of Brain Connectome and Manipulation, the Brain Cognition and Brain Disease Institute (BCBDI), Shenzhen Institute of Advanced Technology (SIAT), Chinese Academy of Sciences, Shenzhen, 518055, China.

^8^ CAS Center for Excellence in Brain Science and Intelligence Technology, Chinese Academy of Sciences, Shanghai, 200031, China.

^#^These authors contributed equally

^*^Correspondence: yj.zhu1@siat.ac.cn

**Supplementary Figures**


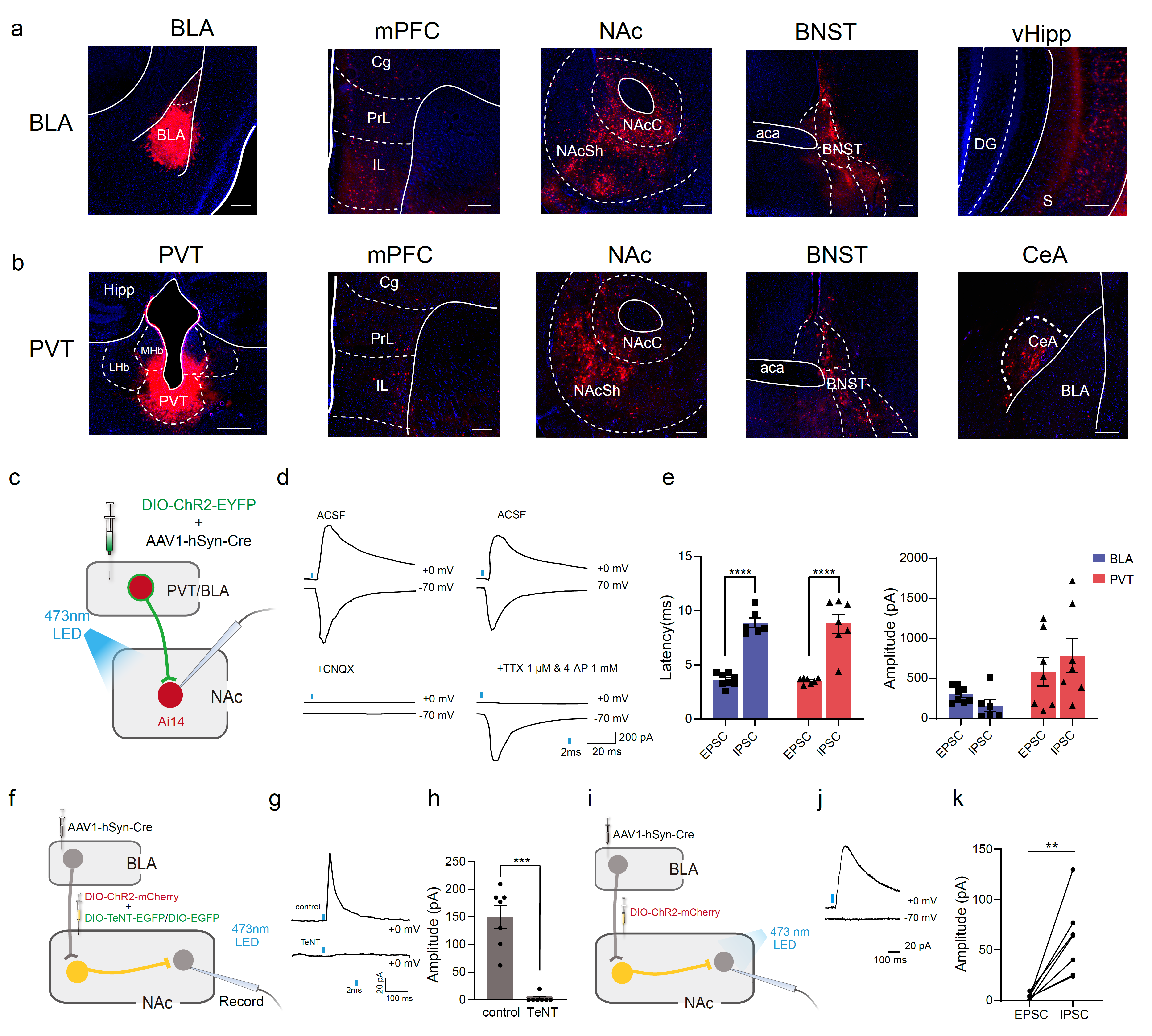


**Supplementary Fig. 1 AAV1-mediated anterograde transsynaptic tagging from the BLA and the PVT**

(**a**) Representative images showing anterogradely labeled neurons in downstream brain regions of the BLA. aca: anterior commissure, anterior part, BLA: basolateral amygdala, BNST: bed nucleus of stria terminalis, Cg: Cingulate cortex, DG: dentate gyrus, mPFC: medial prefrontal cortex, NAc: nucleus accumbens, NAcC: NAc core, NAcSh: NAc shell, S: subiculum, vHipp: ventral hippocampus. Scale bar: 200 μm.

(**b**) Representative images showing and anterograde labeled neurons in downstream brain regions of the PVT. CeA: the central nucleus of the amygdala, lHb: lateral habenula, mHb: medial habenula, PVT: paraventricular nucleus of the thalamus. Scale bar: 200 μm.

(**c**) Schematic showing the experimental design to verify functional synaptic connectivity between BLA/PVT and anterogradely labeled neurons in NAc.

(**d**) Left: Example trace of light-evoked postsynaptic currents with and without CNQX. Right: Example trace of light-evoked postsynaptic currents with and without TTX&4-AP.

(**e**) Latencies and amplitudes of evoked EPSCs recorded from NAc^BLA^ (n = 8 cells) and NAc^PVT^ (n = 7 cells) neurons. Two-way ANOVA: EPSC/IPSC (*F*_(1,24)_ = 110.1, *P* < 0.0001), followed by Sidak’s test , *****P* < 0.0001. Mean ± s.e.m.

(**f**) Schematic showing the experimental design to verify the efficiency of TeNT to block synaptic transmission.

(**g**) Example traces showing light-evoked inhibitory postsynaptic current (IPSC) recorded from neighboring neurons of NAc^BLA^ neurons which did not express ChR2. Scale bar: 20 pA, 100 ms.

(**h**) Amplitude of evoked IPSCs recorded from TeNT group (n = 7 cells) and control group (n = 7 cells). Two-tailed Mann-Whitney test, *P* = 0.0006. Mean ± s.e.m.

(**i**) Schematic showing the experimental design to record postsynaptic currents in neighboring neurons following optical stimulation of NAc^BLA^ neurons.

(**j**) Example trace of light-evoked postsynaptic currents.

(**k**) Amplitudes of light-evoked IPSCs and EPSCs (n = 7 cells). Two-tailed paired t-test, *P* = 0.0060. Mean ± s.e.m.


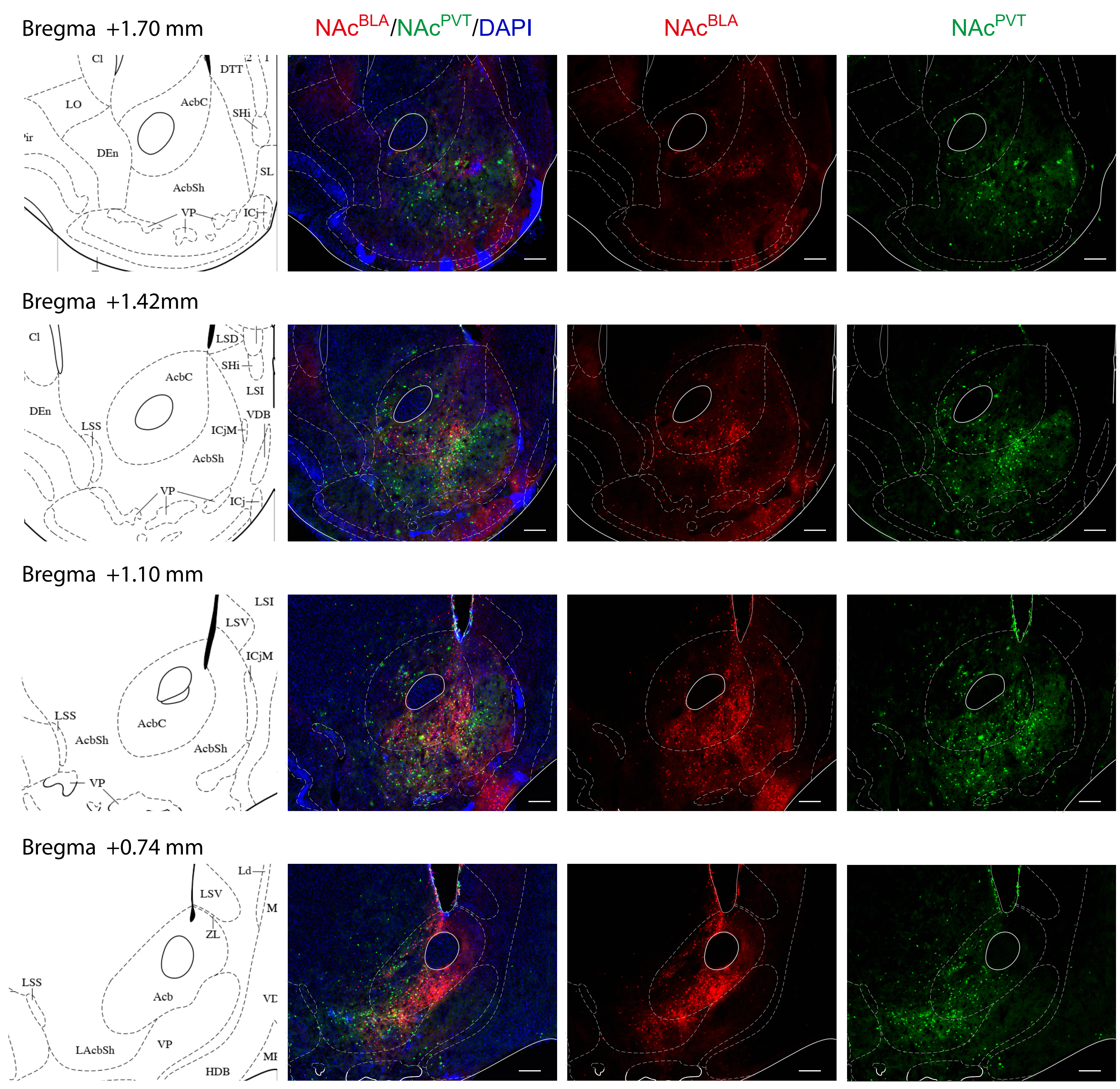


**Supplementary Fig. 2 Distributions of NAc^BLA^ and NAc^PVT^ neurons at different A-P positions.**

Distribution of anterogradely labeled neurons in the NAc receiving BLA (NAc^BLA^ : red) or PVT inputs (NAc^PVT^: green) at different A-P positions. Scale bar: 200 μm.


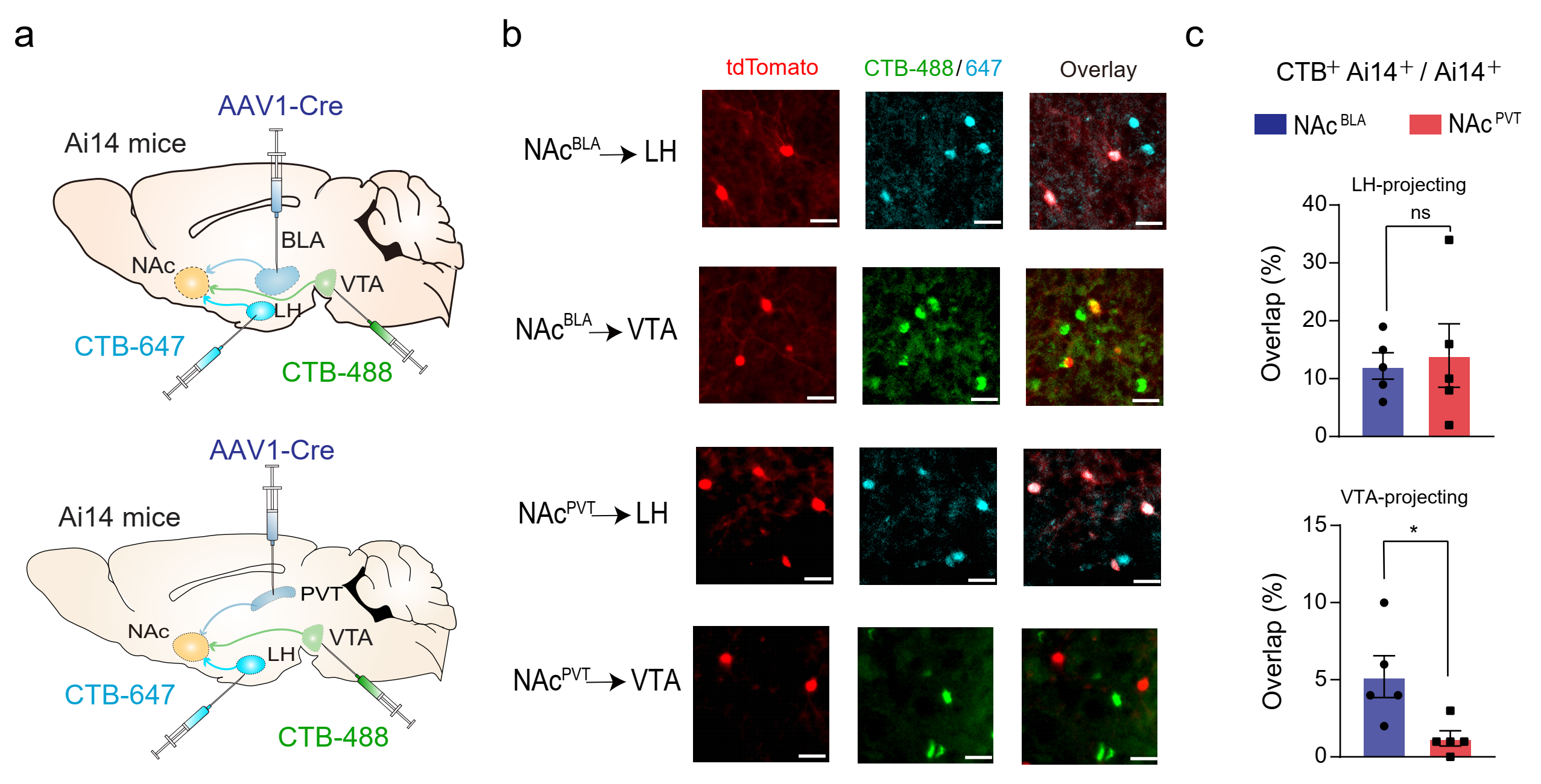


**Supplementary Fig. 3 Overlaps between NAc^BLA^, NAc^PVT^ neurons with LH- or VTA-projecting NAc neurons.**

(**a**) Schematics showing the strategy to reveal the overlap between NAc^BLA^, NAc^PVT^ neurons and LH- or VTA-projecting NAc neurons in Ai14 mice.

(**b**) Example images showing the overlap between NAc^BLA^, NAc^PVT^ neurons with LH- or VTA-projecting NAc neurons. Scale bar: 20 μm.

(**c**) Quantification of the percentage of LH- or VTA- projecting neurons in NAc^BLA^ (n = 5 mice) and NAc^PVT^ (n = 5 mice) subpopulations. Two-tailed Mann-Whitney test. *P* = 0.0159. Mean ± s.e.m.


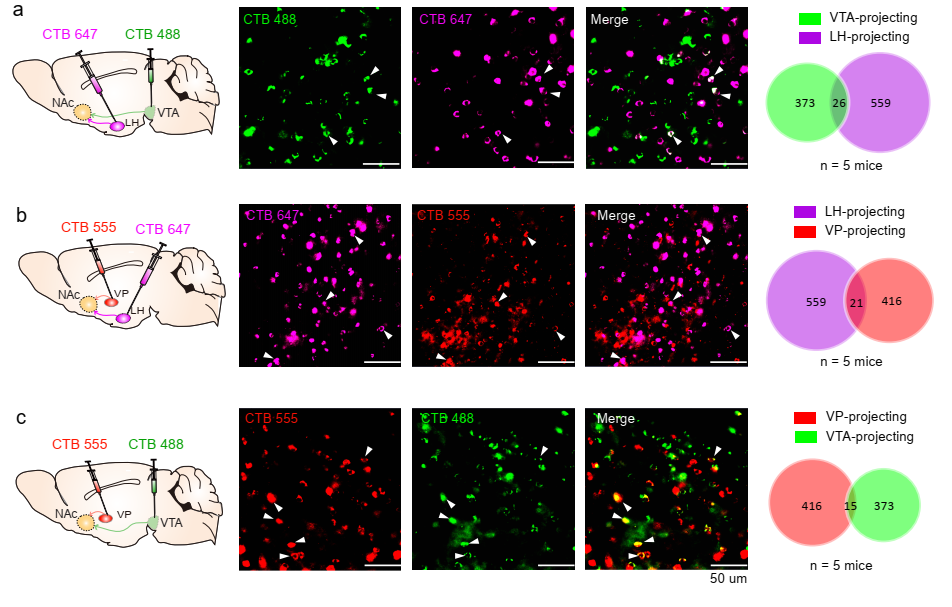


**Supplementary Fig. 4 Overlaps between VP-, LH- and VTA- projecting NAc neurons**

(**a**) Left: Schematics showing the strategy to reveal the overlap between LH- and VTA- projecting NAc neurons. Middle: Example figure. Right: Number and overlap of LH- and VTA- projecting NAc neurons (n = 5 mice);

(**b**) Left: Schematics showing the strategy to reveal the overlap between VP- and LH- projecting NAc neurons. Middle: Example figure. Right: Number and overlap of VP- and LH- projecting NAc neurons (n = 5 mice);

(**c**) Left: Schematics showing the strategy to reveal the overlap between VP- and VTA- projecting NAc neurons. Middle: Example figure. Right: Number and overlap of VP- and VTA- projecting NAc neurons (n = 5 mice);


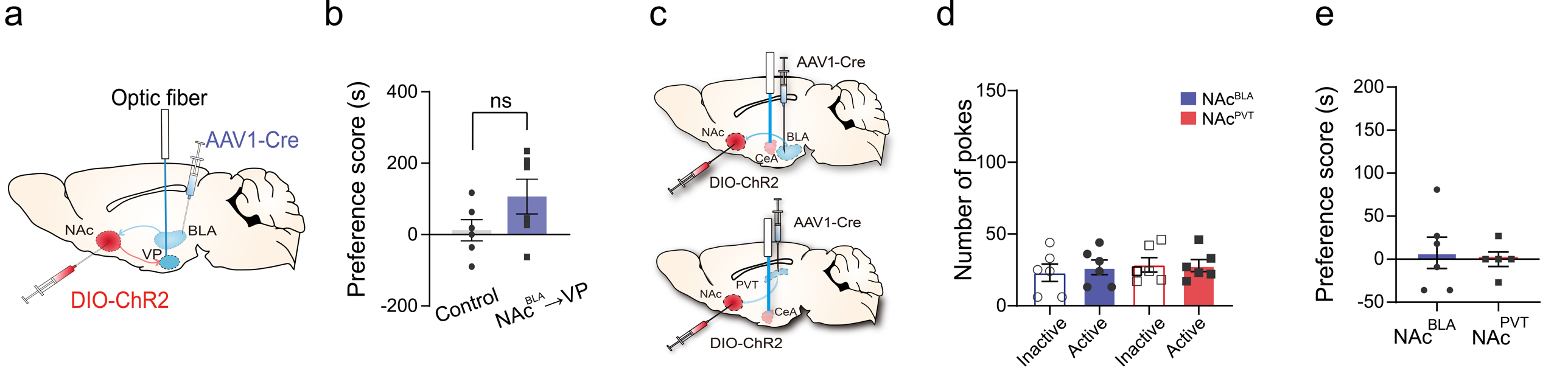


**Supplementary Fig. 5 Activation of the NAc^BLA^🡪VP pathway in the RTPP test.**

(**a**) Schematic showing the experimental design to activate NAc^BLA^ terminals in the VP in the RTPP test.

(**b**) Preference scores in the RTPP test for mice with NAc^BLA^🡪VP pathway activation (n = 6 for each group). Two-tailed Mann-Whitney test. *P* = 0.1797. Ns, not significant. Mean ± s.e.m.

(**c**) Schematic showing the viral strategy in control experiments, to transduce NAc^BLA^ or NAc^PVT^ neurons with ChR2 and to place the optic fiber at CeA.

(**d**) Average numbers of nose pokes for NAc^BLA^ (n = 6) and NAc^PVT^ mice (n = 6). Two-way ANOVA : ChR2 x poke (*F*_(1,10）_ = 0.2241, *P* = 0.6461), poke (*F*_(1,10)_ = 0.8965, *P* = 0.3661), followed by post-hoc Sidak’s test. Mean ± s.e.m.

(**e**) Quantification of the preference score in real-time place preference test for NAc^BLA^::ChR2 (n = 6) and NAc^PVT^::ChR2 (n = 5) mice. Two-tailed one-sample t-test, not significantly different from theoretical mean 0. Mean ± s.e.m.


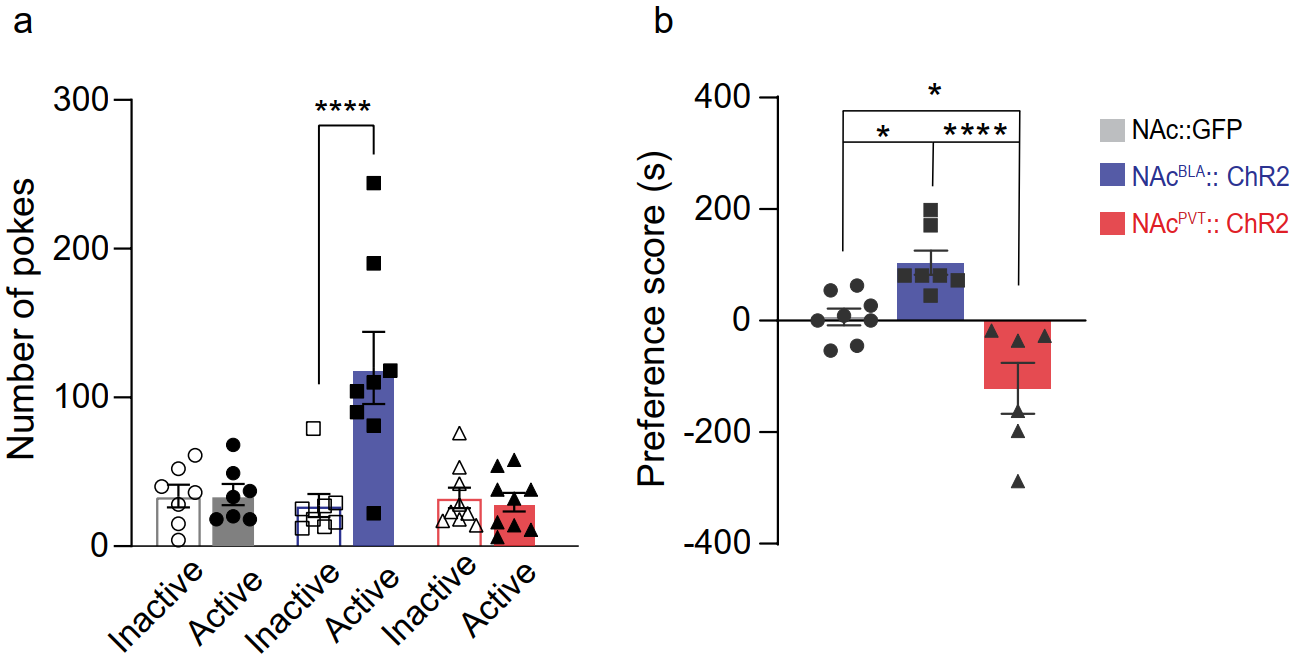


**Supplementary Fig. 6 In female mice, optical stimulation of NAc^BLA^ and NAc^PVT^ neurons induced reward and aversion, respectively.**

(**a**) Average numbers of nose pokes for female NAc::GFP (n = 7), NAc^BLA^::ChR2 (n = 8) and NAc^PVT^::ChR2 mice (n = 9). Two-way ANOVA : ChR2 x poke (*F*_(2,21）_ = 18.03, *P* < 0.0001), poke( *F*_(1, 21)_ = 16.67, *P* = 0.0005 ), ChR2 group (*F*_(2,21)_ = 5.751, *P* = 0.0102), followed by post-hoc Sidak’s test. *****P* < 0.0001. Mean ± s.e.m.

(**b**)Quantification of the preference score in real-time place preference test for female NAc::GFP (n = 8) , NAc^BLA^::ChR2 (n = 7) and NAc^PVT^::ChR2 (n = 6) mice. One-way ANOVA (*F*_(2,18)_ = 15.8, *P* = 0.0001) followed by post-hoc Tukey’s test (NAc^BLA^::ChR2 vs. NAc::GFP, *P* = 0.0449; NAc^PVT^::ChR2 vs. NAc::GFP, *P* = 0.0108; NAc^BLA^::ChR2 vs. NAc^PVT^::ChR2, *P* < 0.0001;). Mean ± s.e.m.


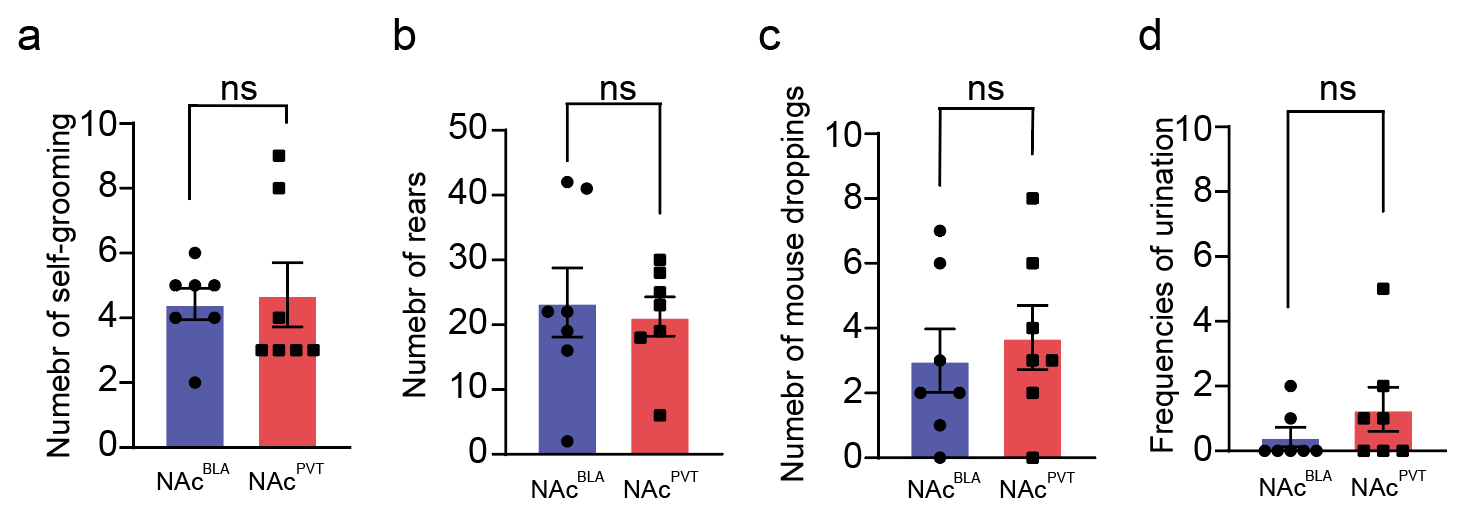


**Supplementary Fig. 7 Microstructures of behaviors in the RTPP test.**

**a,** Average numbers of self-grooming during the RTPP test (n = 7 for each group). Two-tailed Mann-Whitney test. *P* = 0.3963. Ns, not significant. Mean ± s.e.m.

**b,** Average numbers of rears during the RTPP test (n = 7 for each group). Two-tailed Mann-Whitney test. *P* = 0.9172.Ns, not significant. Mean ± s.e.m.

**c,** Numbers of mouse droppings during the RTPP test (n = 7 for each group). Two-tailed Mann-Whitney test. *P* = 0.5268. Ns, not significant. Mean ± s.e.m.

**d,** Frequencies of urination during the RTPP test (n = 7 for each group). Two-tailed Mann-Whitney test. *P* = 0.1795. Ns, not significant. Mean ± s.e.m.


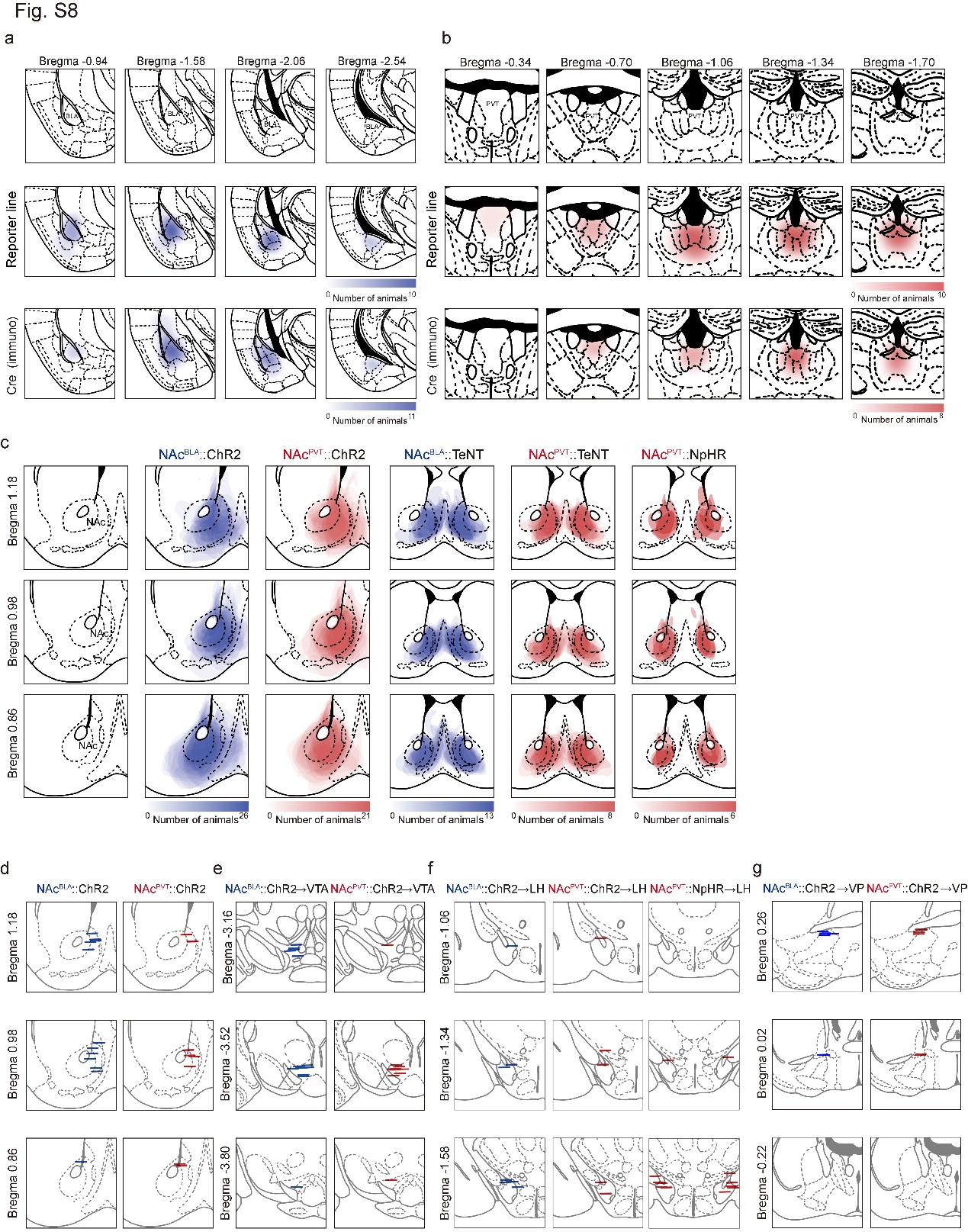


**Supplementary Fig. 8** Locations of virus expression and optic fiber placement for stimulation/ suppression experiments.

**a-b,** Schematics showing viral expression in the BLA and the PVT from anterior to posterior locations, respectively. Schematics in the second row represent injection sites in reporter lines, related to the experiments in Fig. 1 and Supplementary Fig. 2-3. Schematics in the third row represent injection sites in wild type animals in which immunostaining for Cre was performed, related to the experiments in Fig. 2c, 2f, 4b and 4d.

**c,** Schematics showing viral expression for stimulation/ suppression experiments in the NAc from anterior to posterior locations.

**d-g,** Schematics showing optic fiber locations in the NAc, VTA, LH, and VP for stimulation/suppression experiments.
