# Supplementary material for: Reward and aversion processing by input-defined parallel nucleus accumbens circuits in mice
Source: Nat Commun. 2022 Oct 21;13:6244. doi: 10.1038/s41467-022-33843-3 (PMC9587247; doi:10.1038/s41467-022-33843-3)
Supplement: Supplementary file 3 — Description of Additional Supplementary Files [file 41467_2022_33843_MOESM3_ESM.pdf]

Description of Additional Supplementary Files

File Name: Supplementary Code 1
Description: Matlab script
