# Supplementary material for: Reward and aversion processing by input-defined parallel nucleus accumbens circuits in mice
Source: Nat Commun. 2022 Oct 21;13:6244. doi: 10.1038/s41467-022-33843-3 (PMC9587247; doi:10.1038/s41467-022-33843-3)
Supplement: Supplementary file 4 — Supplementary Code 1 [file 41467_2022_33843_MOESM4_ESM.zip › supplementary code/DA example/Instructions.docx]

Analysis of fiber photometry recording using custom written MATLAB codes:

Original data were recorded in TDMS files with THINKERTECH device. Event/trigger data could be store in the same TDMS file as the calcium data or in a separate file with similar file name(x-EVENT), depending on the model of the devices.

A piece of baseline file (recoded in dark, optic fiber not attached to the animal) was recorded before experiments on the same day.

TDMS files can be read with mat files in TDMSReader folder, which is provided by the device company THINKERTECH.

Supporting codes which stores basic functions for calculations and plotting figures were provided in DA example\code\supporting codes

Figures showing the effect of different stimulus frequency, like the Fig. 5e, could be generated with ana_FP_stimDApulses.mat, with the example data ‘signal.tdms’. And the result and plots could be stored in DA example\data\DA folder

Averaged Z-scores of multiple recordings, like Fig. 5b, 5c could be calculated with ana_popu_FP_DA_zscore. This matlab code worked on mat files which stores results generated from ‘ana_FP_stimDApulses.mat’ or similar protocols. In the DA example\data\DA folder, we provided duplicated mat files (signal_ch_ThorCam.mat) which could be used to test this mat code. And the zscores and plots of those example data are stored in the folder data\DA\figure.

If you have any questions with problems in testing the codes, please contact [yj.zhu1@siat.ac.cn](mailto:yj.zhu1@siat.ac.cn) or kk.zhou@siat.ac.cn.
