# Supplementary figures and images for: Reward and aversion processing by input-defined parallel nucleus accumbens circuits in mice
Source: Nat Commun. 2022 Oct 21;13:6244. doi: 10.1038/s41467-022-33843-3 (PMC9587247; doi:10.1038/s41467-022-33843-3)

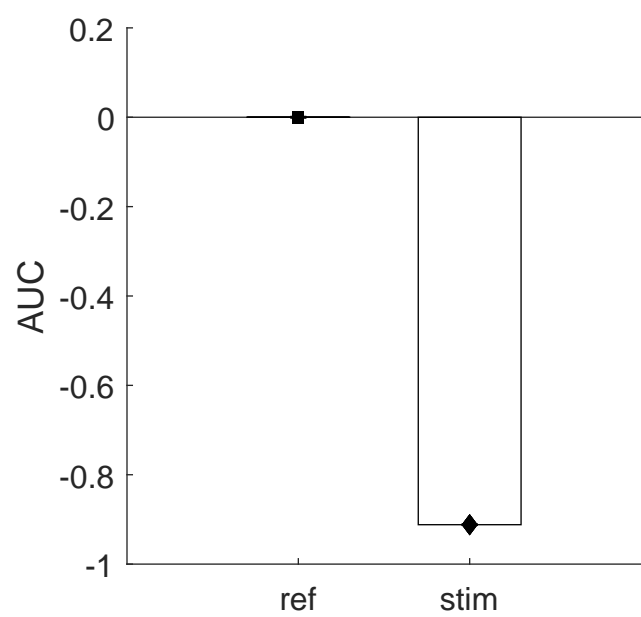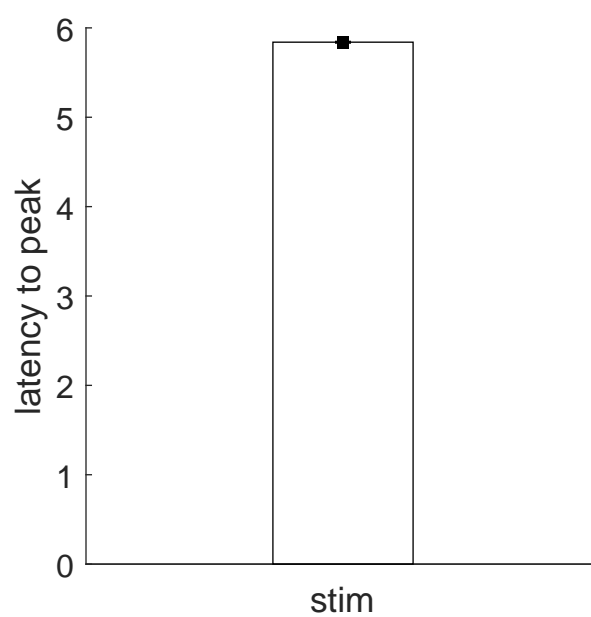

Supplement: Supplementary file 4 — Supplementary Code 1 [file 41467_2022_33843_MOESM4_ESM.zip › supplementary code/DA example/data/DA/figure/DA_HZ_AverageResponse_AUC.pdf]

# DA\_HZ\_AverageResponse

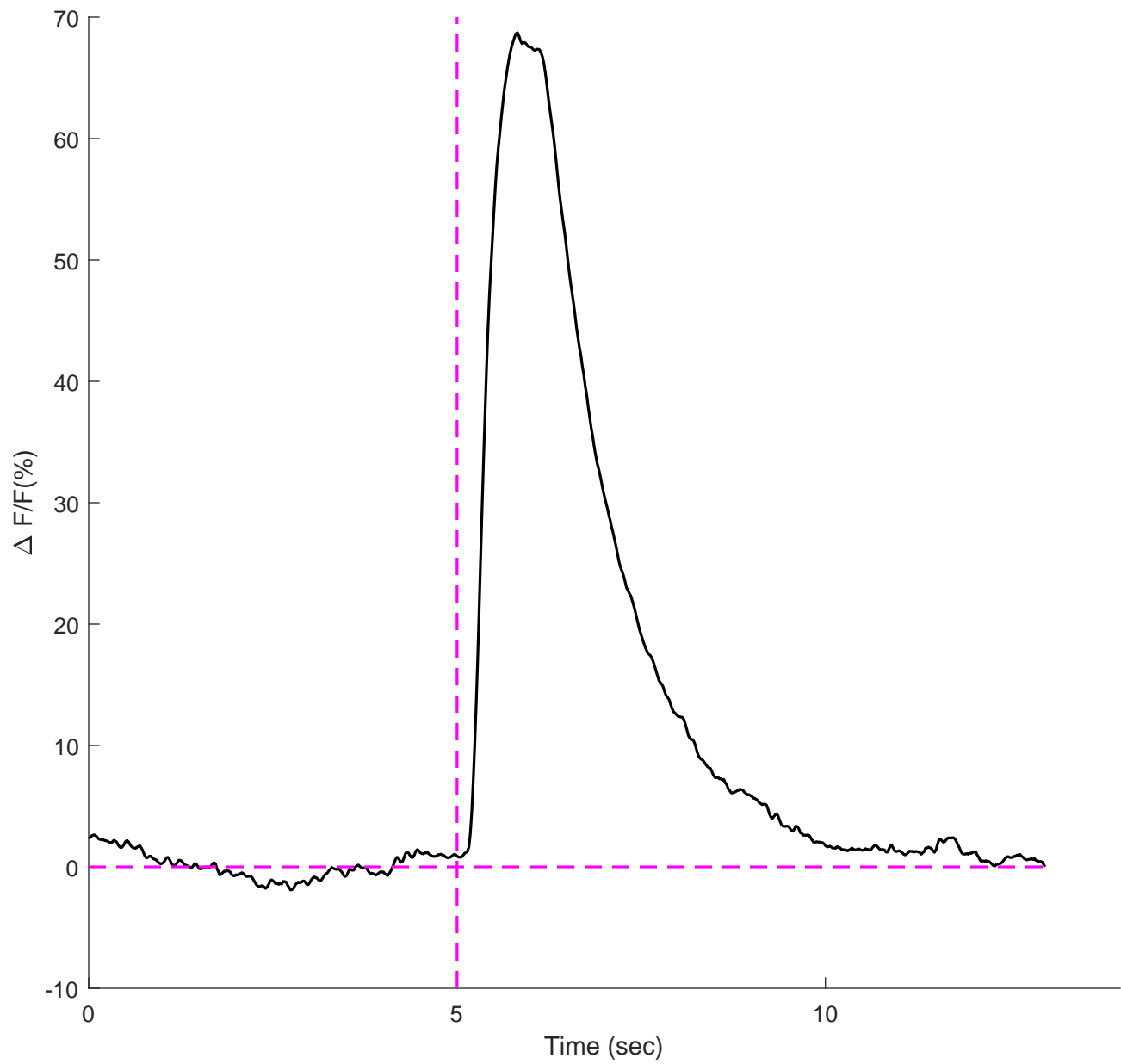

Supplement: Supplementary file 4 — Supplementary Code 1 [file 41467_2022_33843_MOESM4_ESM.zip › supplementary code/DA example/data/DA/figure/DA_HZ_AverageResponse_averge_trace.pdf]

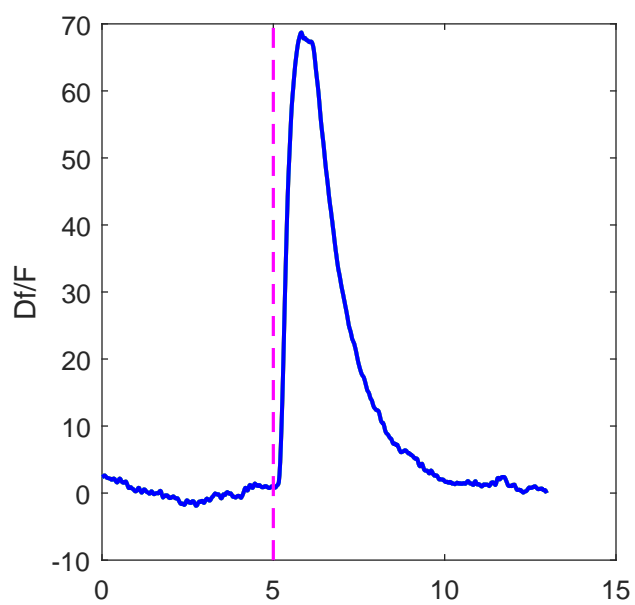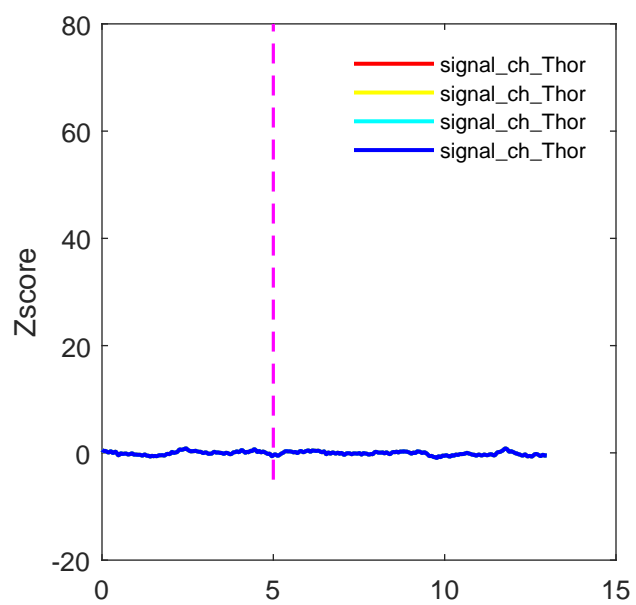

Supplement: Supplementary file 4 — Supplementary Code 1 [file 41467_2022_33843_MOESM4_ESM.zip › supplementary code/DA example/data/DA/figure/DA_HZ_AverageResponse_individual.pdf]

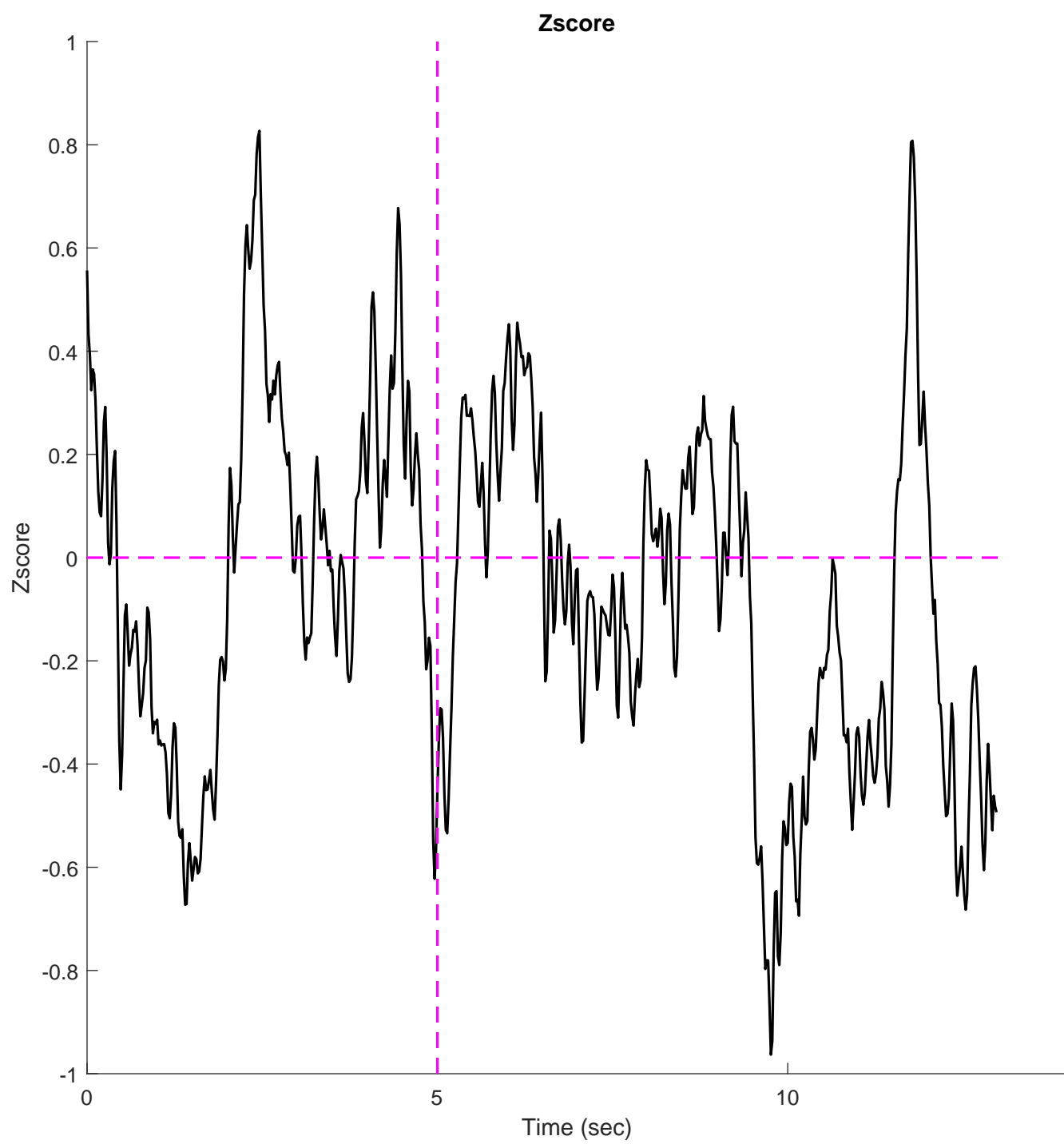

Supplement: Supplementary file 4 — Supplementary Code 1 [file 41467_2022_33843_MOESM4_ESM.zip › supplementary code/DA example/data/DA/figure/DA_HZ_AverageResponse_zscore.pdf]

signal\_ch

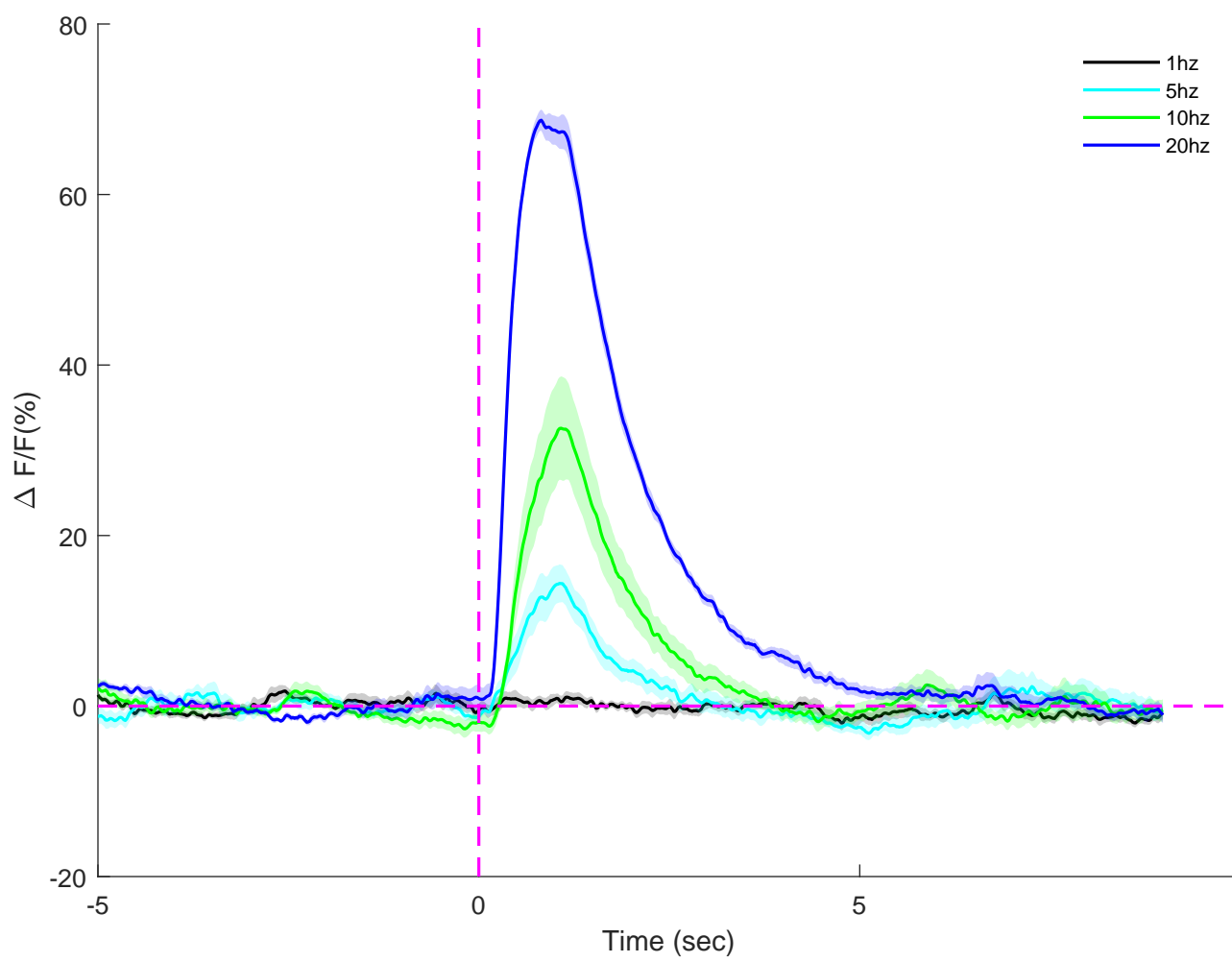

Supplement: Supplementary file 4 — Supplementary Code 1 [file 41467_2022_33843_MOESM4_ESM.zip › supplementary code/DA example/data/DA/signal_ch_fiber_photometry.pdf]

signal\_ch  
Mean heatmap /10 trials

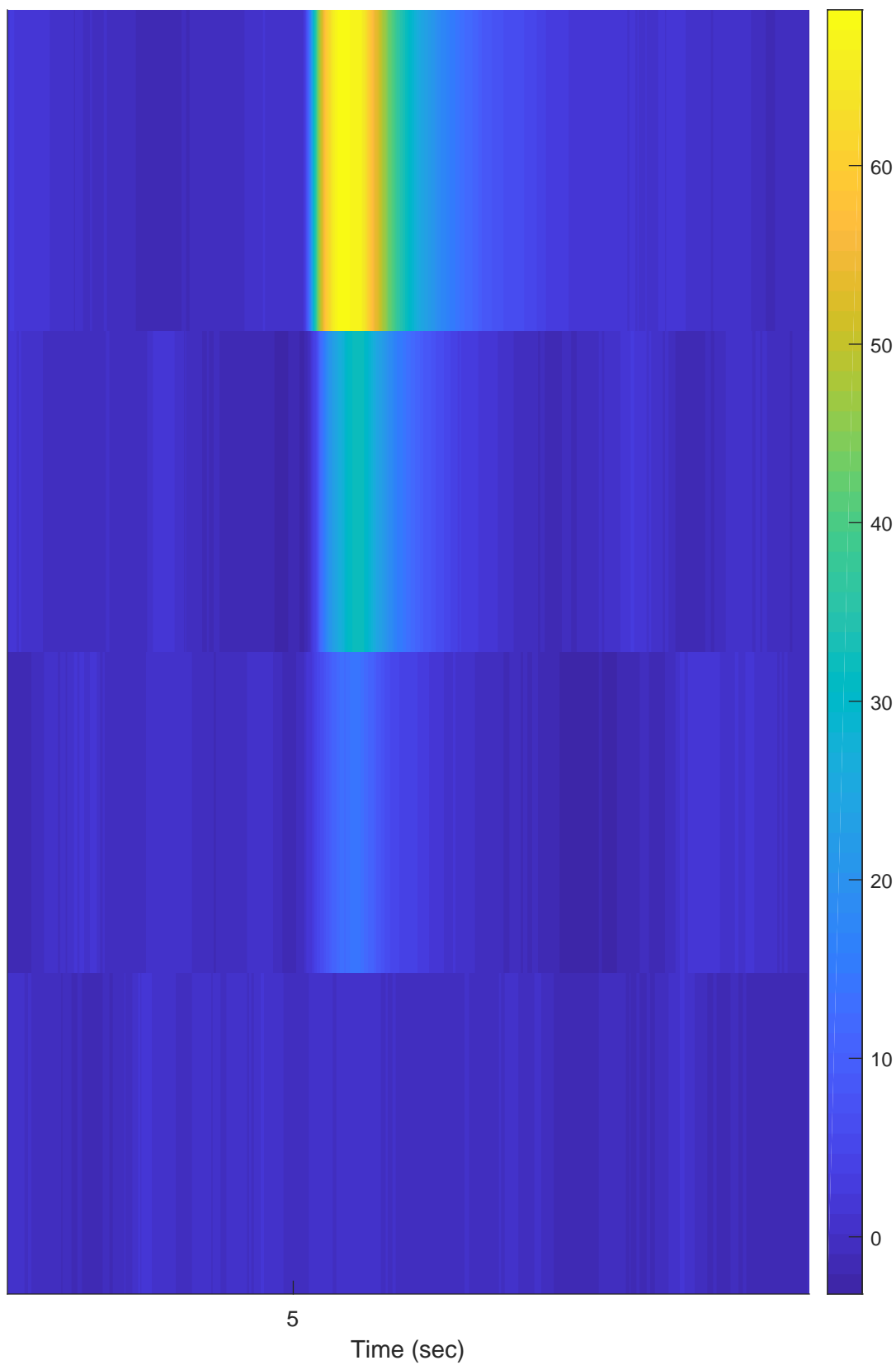

Supplement: Supplementary file 4 — Supplementary Code 1 [file 41467_2022_33843_MOESM4_ESM.zip › supplementary code/DA example/data/DA/signal_ch_Mean_heatmap.pdf]

signal\_ch  
Mean traces /10 trials

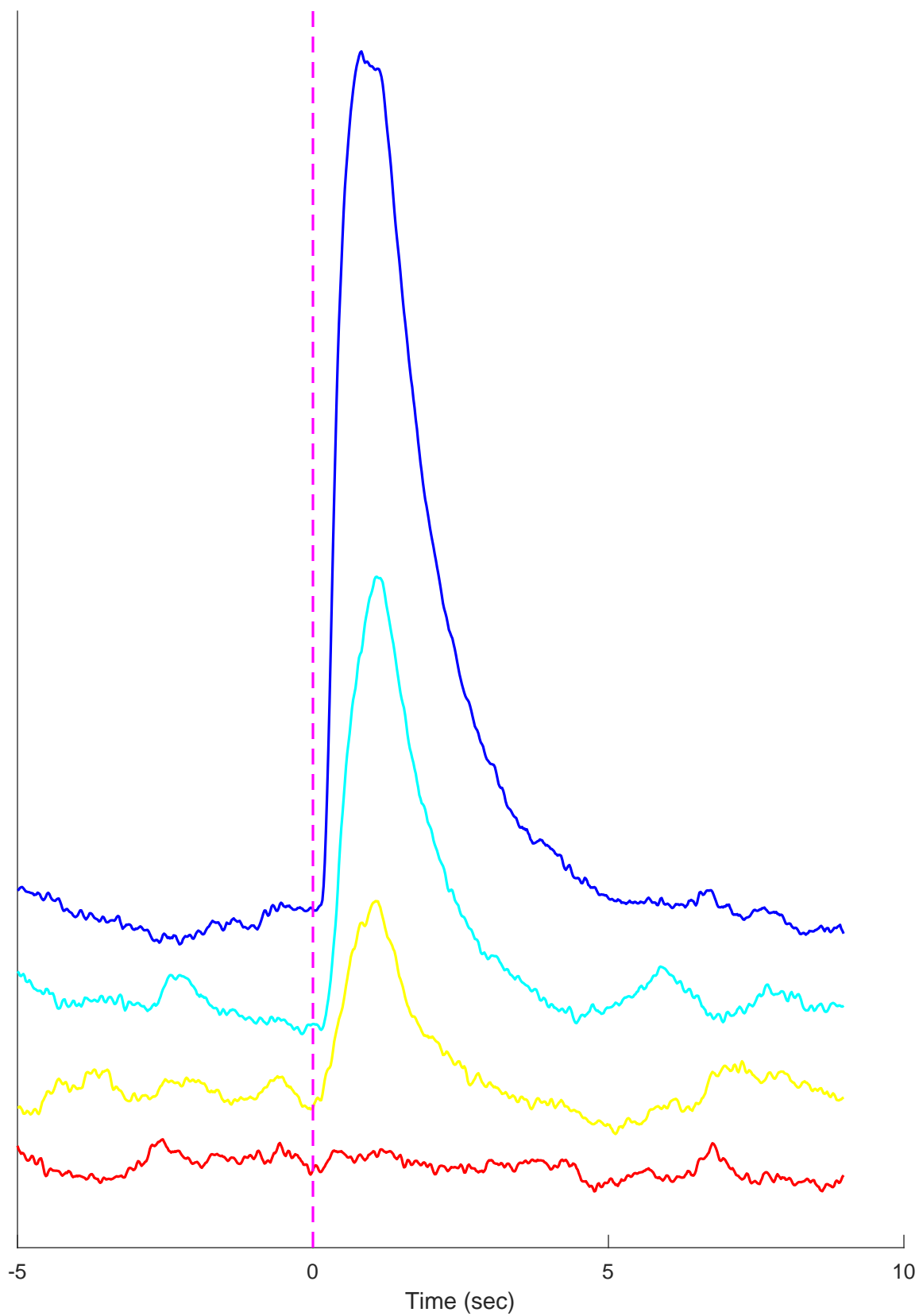

Supplement: Supplementary file 4 — Supplementary Code 1 [file 41467_2022_33843_MOESM4_ESM.zip › supplementary code/DA example/data/DA/signal_ch_Mean_traces.pdf]

signal\_ch

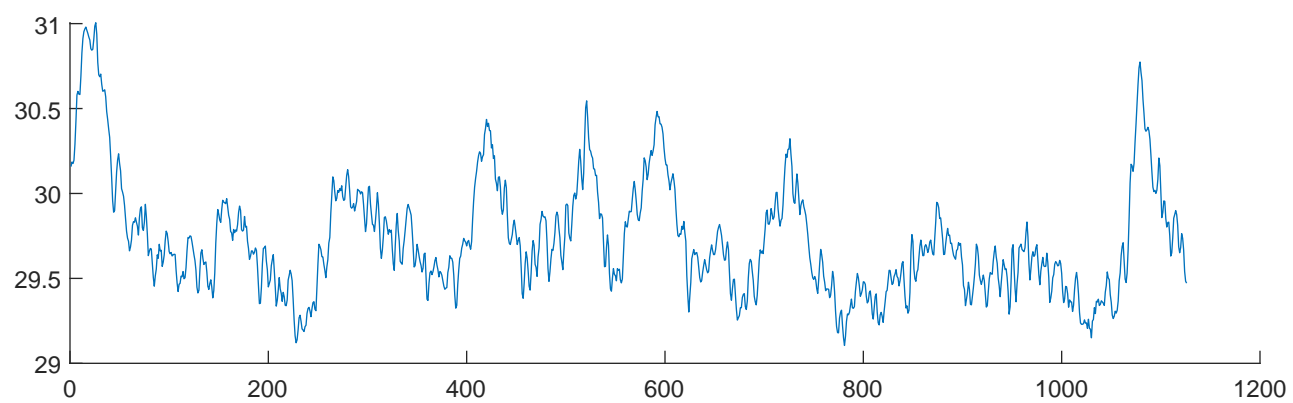

Supplement: Supplementary file 4 — Supplementary Code 1 [file 41467_2022_33843_MOESM4_ESM.zip › supplementary code/DA example/data/DA/signal_ch_raw_traces.pdf]
