# Supplementary material for: Reward and aversion processing by input-defined parallel nucleus accumbens circuits in mice
Source: Nat Commun. 2022 Oct 21;13:6244. doi: 10.1038/s41467-022-33843-3 (PMC9587247; doi:10.1038/s41467-022-33843-3)
Supplement: Supplementary file 5 — Reporting Summary [file 41467_2022_33843_MOESM5_ESM.pdf]

nature portfolio

Corresponding author(s): Yingjie Zhu

Last updated by author(s): Sep 22, 2022

Reporting Summary

Nature Portfolio wishes to improve the reproducibility of the work that we publish. This form provides structure for consistency and transparency in reporting. For further information on Nature Portfolio policies, see our Editorial Policies and the Editorial Policy Checklist.

Statistics

For all statistical analyses, confirm that the following items are present in the figure legend, table legend, main text, or Methods section.

n/a	Confirmed	
☐	☒	The exact sample size (*n*) for each experimental group/condition, given as a discrete number and unit of measurement
☐	☒	A statement on whether measurements were taken from distinct samples or whether the same sample was measured repeatedly
☐	☒	The statistical test(s) used AND whether they are one- or two-sided<br>*Only common tests should be described solely by name; describe more complex techniques in the Methods section.*
☒	☐	A description of all covariates tested
☒	☐	A description of any assumptions or corrections, such as tests of normality and adjustment for multiple comparisons
☐	☒	A full description of the statistical parameters including central tendency (e.g. means) or other basic estimates (e.g. regression coefficient) AND variation (e.g. standard deviation) or associated estimates of uncertainty (e.g. confidence intervals)
☐	☒	For null hypothesis testing, the test statistic (e.g. *F*, *t*, *r*) with confidence intervals, effect sizes, degrees of freedom and *P* value noted<br>*Give P values as exact values whenever suitable.*
☒	☐	For Bayesian analysis, information on the choice of priors and Markov chain Monte Carlo settings
☒	☐	For hierarchical and complex designs, identification of the appropriate level for tests and full reporting of outcomes
☒	☐	Estimates of effect sizes (e.g. Cohen's *d*, Pearson's *r*), indicating how they were calculated

Our web collection on statistics for biologists contains articles on many of the points above.

Software and code

Policy information about availability of computer code

Data collection	Custom written code in Matlab(2020b) was used for the acquisition and analysis of behavioral data from RTPP experiments Zhu, et al., Nature, 2016. Labstate 3.5(Anilab Software and Instruments Co., Ltd) was used for the acquisition and analysis of behavioral data from self-stimulation experiments. ImageJ (v1.52e) was used for analysis of images.
Data analysis	Statistical analyses were performed using Prism 8.0 (GraphPad Software).

For manuscripts utilizing custom algorithms or software that are central to the research but not yet described in published literature, software must be made available to editors and reviewers. We strongly encourage code deposition in a community repository (e.g. GitHub). See the Nature Portfolio guidelines for submitting code & software for further information.

Data

Policy information about availability of data

All manuscripts must include a data availability statement. This statement should provide the following information, where applicable:

- Accession codes, unique identifiers, or web links for publicly available datasets
- A description of any restrictions on data availability
- For clinical datasets or third party data, please ensure that the statement adheres to our policy

Source Data file is provided with the paper. All relevant data supporting the current study are available from the corresponding author upon reasonable request.

Human research participants

Policy information about studies involving human research participants and Sex and Gender in Research.

Reporting on sex and gender	N/A
Population characteristics	N/A
Recruitment	N/A
Ethics oversight	N/A

Note that full information on the approval of the study protocol must also be provided in the manuscript.

Field-specific reporting

Please select the one below that is the best fit for your research. If you are not sure, read the appropriate sections before making your selection.

☒ Life sciences ☐ Behavioural & social sciences ☐ Ecological, evolutionary & environmental sciences

For a reference copy of the document with all sections, see nature.com/documents/nr-reporting-summary-flat.pdf

Life sciences study design

All studies must disclose on these points even when the disclosure is negative.

Sample size	Sample sizes of 5-13 animals were estimated based on previous publications (Zhu, et al., Nature, 2016 and Christoffel et al., Nature Communications, 2021), which were sufficient to determine significance between test and control groups.
Data exclusions	Around 400 mice were used in the current study. Less than 5% of the animals were excluded because of insufficient or inappropriate viral expression. About 1% of animals were excluded because of aberrant behavior noticed before behavioral tests.
Replication	All experiments we performed were reported in the paper. We used female mice to repeat the experiments in Fig. 2c,2f, the results were similar (data shown in Supplementary Fig. 6). Experiments in which representative images were shown in figures 3d, 3j, supplementary figures 1a,b and 2, were repeated independently in at least 5 animals with similar results. And the injection site and viral expression were checked and summarized in supplementary figure8.
Randomization	Animals were housed on an average 5 animal per cage, and were randomly assigned to be in control or test group. In case that multiple behavioral apparatus were used, animals belonged to different cages were randomly assigned to behavioral setups in a counterbalanced manner.
Blinding	All behavioral data were collected in isolated devices with electronic equipments. The investigators were blinded to group assignment during data collection and analysis.

Reporting for specific materials, systems and methods

We require information from authors about some types of materials, experimental systems and methods used in many studies. Here, indicate whether each material, system or method listed is relevant to your study. If you are not sure if a list item applies to your research, read the appropriate section before selecting a response.

Materials & experimental systems

n/a	Involved in the study	
☐	☒	Antibodies
☒	☐	Eukaryotic cell lines
☒	☐	Palaeontology and archaeology
☐	☒	Animals and other organisms
☒	☐	Clinical data
☒	☐	Dual use research of concern

Methods

n/a	Involved in the study	
☒	☐	ChIP-seq
☒	☐	Flow cytometry
☒	☐	MRI-based neuroimaging

Antibodies

Antibodies used	Primary antibody: Anti-GFP, Thermofisher, A11122; Anti-Cre, Millipore, MAB2130. Secondary antibody : Goat anti Rabbit-488 #111-547-003 or Donkey anti Mouse-Cy3,#715-165-150 Jackson Immuno
Validation	Anti-GFP (Thermofisher, A11122), applications: WB, IHC, Flow, Elisa, etc. Host: Rabbit. Manufacture's website: https://www.thermofisher.cn/cn/zh/antibody/product/GFP-Antibody-Polyclonal/A-11122.<br><br>Anti-Cre(MIllipore, MAB3120), applications:ELISA, ICC, IF, IHC, WB, host: Mouse. Website: https://www.merckmillipore.com/CN/zh/product/Anti-Cre-Recombinase-Antibody-clone-2D8,MM_NF-MAB3120?ReferrerURL=https%3A%2F%2Fwww.bing.com%2F&bd=1<br><br>Goat anti Rabbit-488(Jackson Immuno cat#111-547-003),manufacture's website: https://www.jacksonimmuno.com/catalog/products/111-547-003<br>Donkey anti Mouse-Cy3 (Jackson Immuno, cat#715-165-150), manufacture's website:https://www.jacksonimmuno.com/catalog/products/715-165-150

Animals and other research organisms

Policy information about studies involving animals; ARRIVE guidelines recommended for reporting animal research, and Sex and Gender in Research

Laboratory animals	C57BL/6j mice Charles River Laboratories),.GAD2-Cre (JAX Stock No: 010802), DAT-Cre (JAX Stock No: 006660), vGlut2-Cre (JAX Stock No:016963), Ai14 (JAX Stock No: 007908), and R26R-EYFP(JAX Stock No: 006148),GAD67-GFP mice(Dr. Nobuaki Tamamaki's lab) and FSF-tdTomato (Dr. Z. Josh Huang's lab) were used in the experiments. Cre/Flp double reporter mice were obtained by crossing R26R-EYFP mice with FSF-tdTomato mice. All experiments were performed on mice aged 5-12 weeks in the study.
Wild animals	Not involved.
Reporting on sex	Data in main figures were collected in male mice. Following the suggestions from the reviewer, we have also test female mice in the self-stimulation and RTPP tests, and those data were presented in the supplementary figure 6. The data from female mice also support our conclusion on the functional divergence between NAcBLA and NAcPVT neurons.
Field-collected samples	Not involved.
Ethics oversight	All animal husbandry and experimental procedures in the current study were approved by the Animal Care and Use Committees at the Shenzhen Institute of Advanced Technology (SIAT), Chinese Academy of Sciences (CAS).

Note that full information on the approval of the study protocol must also be provided in the manuscript.
